# The Multiple Roles of Periostin in Non-Neoplastic Disease

**DOI:** 10.3390/cells12010050

**Published:** 2022-12-22

**Authors:** Lina Yang, Tongtong Guo, Yuanyuan Chen, Ka Bian

**Affiliations:** 1 College of Life Science, Northwest University, Xi’an 710069, China; 2 Department of Otorhinolaryngology, Tangdu Hospital, Fourth Military Medical University, Xi’an 710032, China

**Keywords:** periostin, variants, non-neoplastic diseases, roles, biomarker, drug

## Abstract

Periostin, identified as a matricellular protein and an ECM protein, plays a central role in non-neoplastic diseases. Periostin and its variants have been considered to be normally involved in the progression of most non-neoplastic diseases, including brain injury, ocular diseases, chronic rhinosinusitis, allergic rhinitis, dental diseases, atopic dermatitis, scleroderma, eosinophilic esophagitis, asthma, cardiovascular diseases, lung diseases, liver diseases, chronic kidney diseases, inflammatory bowel disease, and osteoarthrosis. Periostin interacts with protein receptors and transduces signals primarily through the PI3K/Akt and FAK two channels as well as other pathways to elicit tissue remodeling, fibrosis, inflammation, wound healing, repair, angiogenesis, tissue regeneration, bone formation, barrier, and vascular calcification. This review comprehensively integrates the multiple roles of periostin and its variants in non-neoplastic diseases, proposes the utility of periostin as a biological biomarker, and provides potential drug-developing strategies for targeting periostin.

## 1. Introduction

Periostin (encoded by the *Postn* gene) was first recognized in 1993 from a mouse osteoblastic cell line, a secreted molecule containing no transmembrane domain, and was initially known as osteoblast-specific factor-2(OSF-2) [1]. It was renamed periostin in 1999 due to its preferential expression in the periosteum and periodontal ligament in adult mice reported by Horiuchi et al. Periostin used as a regulator promotes the adhesion and differentiation of osteoblasts [2].

**Figure 1:**
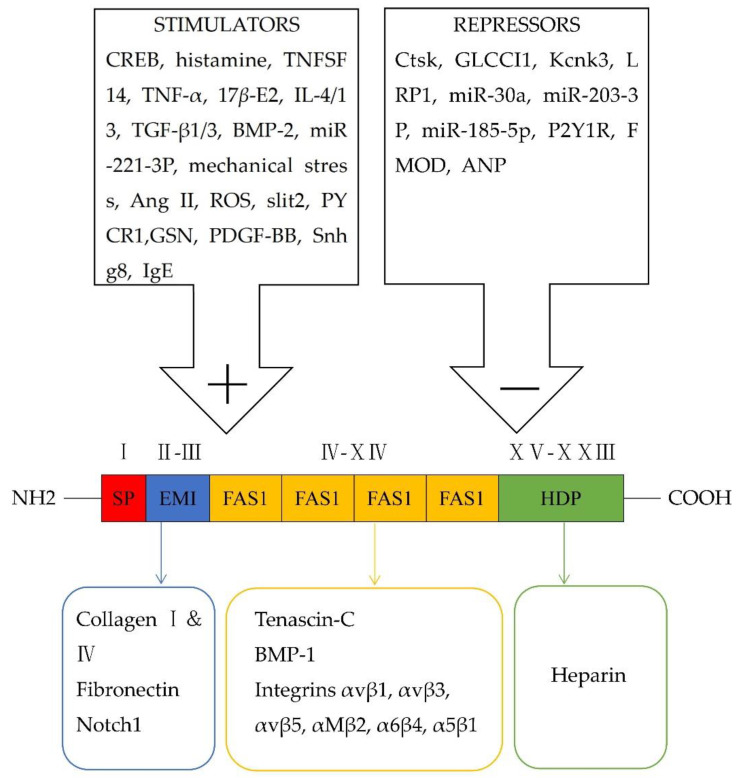
Schematic representation of modular structural domains of periostin and its interaction with different receptors, as well as its stimulators and repressors.

**Figure 2:**
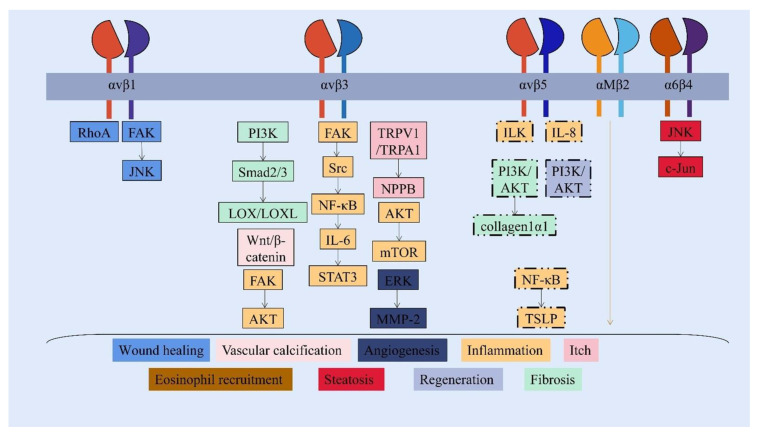
Periostin-integrins interaction and activation of downstream signaling pathways. The FAS1 domain interacts with integrin receptors to activate different and overlapping signaling pathways, which modulate the progression of non-neoplastic diseases under pathological status. (Shared signaling pathways of integrin αv are shown in the box with a dotted line.)

**Figure 3:**
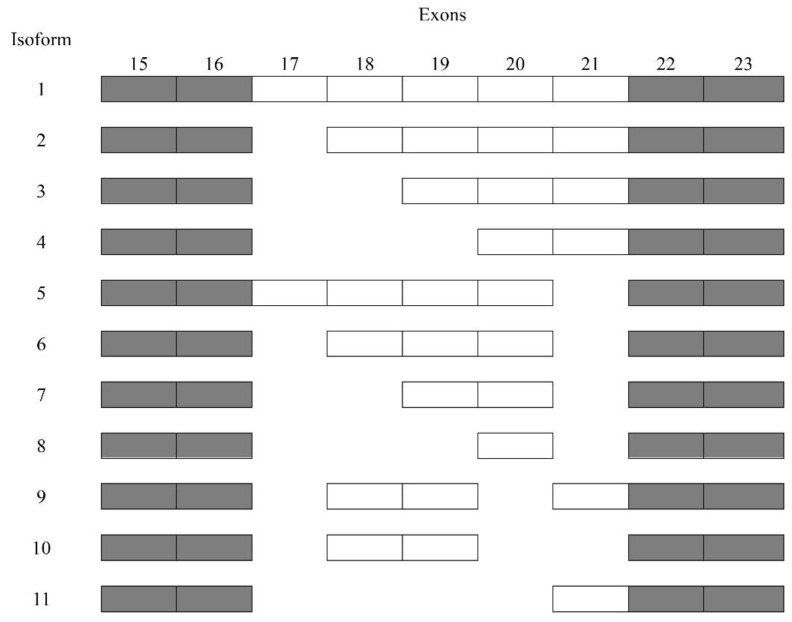
Sequencing of periostin splice variants.

At approximately 90 kDa, periostin as an N-glycoprotein contains 23 exons exhibiting an NH2-terminal secretory signal peptide, accompanied by a highly conserved cysteine-rich EMI domain, which engages in the formation of multimers through cysteine disulfide bonds [3,4], four consecutive and homologous tandem Fasciclin I (FAS1) domains binding to integrins (αvβ1, αvβ3, αvβ5, αMβ2, α6β4, α5β1) and a COOH-terminal hydrophilic domain as an alternatively spliced region consisting of exons 15–23 (Figure 1) [5,6,7,8,9]. Periostin-integrin interactions lead to the activation of signaling pathways (Table 1; Figure 2). Apart from interacting with integrin receptors, it also binds other ECM proteins, for example, collagens, fibronectin, tenascin C, or heparin [4,10]. Periostin can form 11 splice variants (Figure 3). The expression pattern of periostin splicing variants has been reported in cerebral ischemia, asthma, MI, IPF, retinal ischemia, pIBD, joint, and serum (Table 2). Comparability of mouse and human periostin amino acid is 89.2% overall and 90.1% in a mature condition. Mouse and human periostin are respectively located on chromosome 3 and chromosome 13q.

Periostin is commonly overexpressed in human tissues during pathological processes. Periostin, as a matricellular protein and an ECM protein, exerts different roles in tissue development and progression of diseases, including brain injury, ocular diseases, chronic rhinosinusitis, allergic rhinitis, dental diseases, atopic dermatitis, scleroderma, eosinophilic esophagitis, asthma, cardiac diseases, lung diseases, liver diseases, chronic kidney diseases, inflammatory bowel disease, and osteoarthrosis. In a normal physiological situation, periostin is beneficial in mediating teeth development, maintaining the integrity of periodontal ligament (PDL) in postnatal teeth enamel formation, and mediating bone remodeling after orthodontic movement [119,120]; periostin promotes migration of mesenchymal cells in an αvβ3- and β1-based Rho/PI3K signaling mechanism during valve maturation [10]. Besides, during pathogenesis, the roles of periostin are more extensive, including tissue remodeling, fibrosis, inflammation, wound healing, repair, angiogenesis, tissue regeneration, bone formation, barrier, and vascular calcification; this makes it different from other ECM proteins.

Periostin assists in modulating the ECM network [9]. Periostin/BMP-1/LOX cascade assisted in collagen cross-linking [121]. During abnormal scar formation, periostin stimulated the secretion of TGF-β1 via the RhoA/ROCK signaling pathway in human dermal fibroblasts (HDFs), yielding a vicious circle [122].

Herein, we have sufficient knowledge of its stimulators, repressors, expression levels, expression patterns, and roles, as well as periostin-involved signaling pathways, the potential of its guiding choice for medicine, and the serviceability of it as a prospective marker in various diseases. Developing drugs based upon periostin-involved functions or certain periostin isoforms-mediated distinctive roles is beneficial in relieving diseases.

## 2. Early Brain Injury (EBI) and Cerebral Ischemia

The periostin was upregulated in neurons and capillary endothelial cells in the cerebral cortex at 24 h post-subarachnoid hemorrhage (SAH) and initiated BBB disruption, possibly via p38/ERK/MMP-9 signaling pathways and induction of tenascin-C [11].

Following transient cerebral ischemia, isoform 2 minimized the area of cerebral infarction displaying a neuroprotective role with phosphorylation of Akt [111]. Greater serum periostin levels were related to a larger cerebral infarction area and more serious neurological defects at 6-28 days following ischemia [14]. Toll-like receptor 4 (TLR4) selective blockade-IAXO-102 and clarithromycin inhibited BBB disruption and periostin expression [12,13].

## 3. Cardiovascular Diseases

### 3.1. Myocardial Infarction (MI)

Ang II evidently increased periostin through Ras/p38 MAPK (mitogen-activated protein kinase)/CREB and ERK/TGF-β1 pathways in myocytes and fibroblasts [123]. Detection of human tissue specimens reflected prominently high periostin expression in ischemic and reperfused tissue, as well as no expression in healthy myocardium [59]. The lineage analyses of mice verified that periostin-expressing CFs mainly derived from a mass of TCF21^+^ cells [124]. After MI, TGF-β1, mechanical pressure, and Cyclic AMP response element-binding protein 1 (CREB) stimulated cardiac fibroblasts, thereby augmenting ECM deposition, development of collagenous scar and cardiac remodeling, and release of periostin [125]. TGF-β1 upregulated periostin levels in CFs and vascular smooth muscle cells (VSMCs) employing Smad signaling pathways [126,127]. Periostin showed minimal levels under miR-203-3p-binding circumstances restricting cardiomyocytes apoptosis. However, the complex of periostin, miR-203-3p, and small nucleolar RNA host gene 8 (Snhg8) mediated neonatal mouse cardiomyocytes (NMCMs) apoptosis after hypoxia-treated NMCMs, contributing to acute myocardial infarction [66]. Treatment of MI with cardiac mesenchymal stem cells (MSCs) marked by Nestin demonstrated a greater effect on cardiac healing than bone marrow-derived MSCs (NesbmMSCs), which results from part involvement of periostin-induced M2 macrophage polarization [65]. In a rat MI model, Yoshiaki Taniyama et al. discovered four periostin isoforms, including isoforms 1, 2, 5, and 6. Isoform 1 decreased the attachment of fibroblasts and myocytes as well as facilitated myocyte death, leading to ventricular dilation and tissue remodeling. Blockade of exon 17 as prior target assists in protesting cardiac remodeling, diminishing fibrosis, ameliorating ejection fraction, and cardiac function eight weeks after MI [114]. Isoform 6 can mediate the migration of activated fibroblasts and the healing of impaired tissue by αv/FAK/AKT cascade [59]. The inhibition of periostin by valsartan might have an improved effect on cardiac remodeling after MI [57].

### 3.2. Cardiomyocyte Regeneration

Release of periostin facilitated cardiomyocyte regeneration and angiogenesis by interacting with αvβ1, αvβ3, or αvβ5 integrins on myocytes and vascular endothelial cells to activate the PI3K-Akt pathways after MI. The treatment of animals with periostin patches (lacking the N-terminal signal peptide and C-terminal region) not only perfected cardiac fraction and ejection fraction but also contained fibrosis after MI [5]. Periostin eased inflammation and induced reentry of the cardiomyocytes cycle via TNF-α/NF-κB signaling transduction in conjunction with a declining caspase 7 activity [67]. Periostin ablation hindered myocardial regeneration by suppressing the PI3K/AKT/cyclin D1 transmission [68]. Another work in a mouse model of overexpressed full-length periostin indicated that periostin did not speed up the DNA synthesis of cardiomyocytes [128]. Further studies are needed to clarify these issues.

### 3.3. Heart Failure

In diabetic rat hearts, periostin is noticeably overexpressed relative to healthy controls [79]. In the experimental autoimmune myocarditis (EAM) rats model, periostin was spotted in macrophages and fibroblasts. It elicited cardiac fibrosis, likely by recruiting immune cells [60]. A recent examination of atrial appendages from atrial fibrillation (AF) patients suggested a clear association between periostin levels of atrial tissues and deteriorated heart failure, as well as lessened ejection fraction [129]. MiR-30a and fibromodulin (FMOD) tempted the descent of periostin levels and the decrease of atrial fibrosis [61]. GSN, silencing P2Y1R, and slit2-Robo1 pathways inversely initiated periostin release, tempting fibrosis [62]. Periostin prompted pyroptosis by triggering the NLRP3/caspase-1 pathway during myocardial ischemia-reperfusion injury (MIRI) [56].

Valsartan and simvastatin (SIM) hindered periostin expression and alleviated pathologic remodeling [63,79]. Targeting of diabetic animals with the antioxidant resveratrol limited myofibroblast activation and downregulated the expression of periostin via suppressing ERK/TGF-β signaling [69].

### 3.4. Valvular Heart Disease (VHD)

Periostin expression intensively goes up in valvular interstitial cells (VICs) of the mitral valve, compared to wild-type mice. The mitral valve biopsies of male patients going through prosthetic surgery detected a pronounced enhancement in periostin in the ventricular [130]. Besides, periostin was firmly upregulated in the infiltrated inflammatory cells and myofibroblasts within patients with atherosclerotic or rheumatic valves. Meanwhile, massive periostin in the valve leaflet brings about extensive production of matrix metalloproteinase-2 (MMP-2) and MMP-9, leading to severe fibrosis in atherosclerotic and rheumatic VHD [64]. Periostin also prompted the osteogenic potential of aortic valve calcification [131].

### 3.5. Hypertension and Vascular Calcification

Atrial natriuretic peptide (ANP) inhibited periostin expression in the VSMCs and cardiac fibroblasts [70], but oxidative stress contributes to periostin production [132]. The increase in periostin augmented the differentiation and migration of VSMCs [133].

In a hyperlipidemia-associated model of rats, periostin upregulation caused calcium deposits through the successive inhibition of p53 and SLC7A11 in VSMCs [71]. Additionally, plasma periostin levels were positively connected with the Agatston score in patients with coronary artery calcification (CAC). Periostin promoted glycolysis and mitochondrial malfunction as well as contained peroxisome proliferation-activated receptor γ(PPARγ) in VSMCs, thereby provoking arterial calcification [58].

## 4. Ocular Diseases

IL-13 obviously stimulated periostin in conjunctival fibroblasts and, to a much smaller extent, in conjunctival epithelial cells. The recruitment of eosinophils and Th2 cytokines expression, including CCL5, IL-4, and IL-13, were restricted in periostin-deleted AC mice [15]. The concentration of tear periostin is heavier among patients with atopic keratoconjunctivitis (AKC) relative to healthy controls. Tear periostin levels had an infinitely positive association with complications of AKC by acting on corneal or conjunctival epithelial cells [17]. Tear periostin was decreased by treating with tacrolimus or betamethasone along with ameliorative clinical traits in the majority of patients with AKC [16].

Periostin upregulation may assist in scleral remodeling in myopia [18]. It was also manifestly increased in the vitreous of patients with proliferative vitreoretinal diseases, such as proliferative vitreoretinopathy (PVR) and proliferative diabetic retinopathy. It was colocalized with α-SMA and M2 macrophage markers in the retinal fibrovascular membrane (FVM). The inhibition of it decreased retinal FVM formation [134]. Another study of diabetic retinopathy patients uncovered a positive correlation between serum periostin with continuous retinopathy and FVM formation [19]. Expression levels of isoforms 1, 2, and 5 are increased when the preretinal pathological neovascularization (NV) reaches the peak; they may be specific periostin splice variants for preretinal pathological NV in retinal ischemia [116].

## 5. Dental Diseases

During early periodontitis, Wnt5a/CaMKII/Periostin axis mediated collagen and bone formation, maintaining periodontal stabilization [135]. Applying gingivectomy to a rat model presented that periostin promotes ECM generation, as well as increases the formation of fibronectin and collagen via β1/FAK (focal adhesion kinase)/JNK propagation during wound healing. Periostin is not related to myofibroblast differentiation accounting for lessened scar generation [31]. By introducing an excisional palatal model, periostin mRNA and protein expression were upregulated, and it is correlated with fibronectin generation, transition to myofibroblast, and attachment of macrophages to the wound region. Periostin modulated palatal healing via the integrinβ1/RhoA pathway [31].

IL-4 and IL-13 evidently stimulated periostin expression in the human PDL (hPDL). HPDL cells displayed increased proliferation and migration and no significant difference in the generation of inflammatory cytokines under periostin stimulation [34,136]. TNF-α/periostin/JNK promoted the adhesion and osteogenic differentiation competence of human periodontal ligament stem cells (PDLSCs) [34,35].

GCF periostin levels are degressive with the activity and severity of periodontal disease, suggesting its beneficial role in maintaining the function of normal periodontal tissue [32,33]. Salivary periostin levels are positively linked to gingival inflammation and aggressive periodontitis (AgP) severity [33].

## 6. Chronic Rhinosinusitis (CRS) and Allergic Rhinitis (AR)

Among patients of CRS with nasal polyposis (CRSwNP), expression of the periostin gene seemed to be notably upregulated in nasal polyps than in normal sinus mucosa [137]. Mi1on’ski et al. revealed upregulation of periostin in non-polyp and polyp tissue of patients with CRS compared with patients without CRS [138].

CRSwNP and AR were taken for Th2-dominant inflammatory diseases. Higher periostin levels were related to increased basement membrane thickness, subepithelial fibrosis, and eosinophilia among patients undergoing surgery for CRS [20]. Periostin-induced tissue remodeling by activating the Src/AKT/mTOR signaling pathway and inducing myofibroblasts differentiation and expression of ECM or by enhancing the mRNA expression of MMP-3, MMP-7, MMP-8, and MMP-9 in fibroblasts and MMP-9 in epithelial cells in CRS. Additionally, IgE enhanced the periostin expression by a cultured human mast cell line (LAD2 mast cells), thereby leading to epithelial cells secreting thymic stromal lymphopoietin (TSLP) by binding to integrin, in turn activating mast cells to produce IL-5 [24]. Glucocorticoids (GCs) eased CRS by restricting the increase of periostin [28]. Previous studies thought that tissue periostin expression has evident relation with IL-5 and IL-13 levels among patients with CRSwNP [139,140].

A controversial finding demonstrated that the complete absence of periostin might result in mast cell attachment and polyp-like signs in CRSwNP [25]. Serum periostin was higher in patients with CRS than in controls [141]. Periostin levels of nasal lavage fluids (NLF) might function as a reliable marker involved in CRS [27]. Application of omalizumab, mepolizumab, methylprednisolone, and doxycycline into CRSwNP subjects arrested periostin production and inflammatory responses. Doxycycline decreased nasal periostin levels (*p* = 0.084), leading to the less frequent onset of asthma and reduced relapse of nasal polyps [22].

In an ovalbumin-treated murine model of AR, periostin knockout mice appeared to have lesser eosinophils, lower nasal symptom scores, and minimal nasal remodeling than controls [21]. Serum periostin serves to estimate the clinical responses to sublingual immunotherapy (SLIT) within house dust mite (HDM)-induced AR subjects [23]. Asarum heterotropoides (AH) and Angelica gigas extract (AG-Ex) interfered with periostin release in HNEpCs (human nasal epithelial cells) and alleviated AR symptoms [29,30]. The treatment of AR with nasal neurectomy pronouncedly reduced NLF periostin value [142].

## 7. Asthma

Allergic asthma is mostly a Th2-involved heterogeneous inflammation attended by eosinophilia, airway hyperresponsiveness (AHR), and excessive mucus secretion from goblet cells. IL-13/IL-4 have been found to induce periostin expression in bronchial epithelial cells and lung fibroblasts. Periostin isoforms 6, 7, and 8 are evidently expressed in lung fibroblasts [112]. MiR-185-5p negatively modulates mRNA and protein expression of periostin within airway cells and sputum periostin concentration [143]. MiR-221-3p provoked airway eosinophilic inflammation by suppressing CXCL17 expression and subsequently upregulating CCL24, CCL26, and periostin expression in HDM-stimulated mice [144].

A previous investigation into the aspergillus fumigatus antigen-challenged mice model supported that periostin serves a beneficial role in protesting AHR, serum IgE levels, and outcome of peribronchial fibrosis by intensifying TGF-β-mediated Treg differentiation [51]. Besides, another mouse model revealed that periostin suppressed mucus production of goblet cells and increased airflow by checking the expression of Gob5 and Muc5ac [52]. The roles of periostin absence in goblet cell metaplasia (GCM) were involved in at least two pathological mechanisms: direct impacts on differentiation of airway epithelial cells to goblet cells and indirect influences by changing the number of DC-derived cytokines acting on T cells.

A study in HDM-challenged mice offered the opposite effect. Periostin-expressing dendritic cells (DCs) from HDM-challenged wild-type mice kept asthma-like features and IL-13 responses after transferring into periostin null mice [43]. Application of anti-periostin antibody OC-20 weakened the AHR, IgE response, IL-13 responses, and DNA synthesis of T cells incubated with periostin-positive DCs. Periostin-overexpressed epithelial cells manifested that release of TGF-β in epithelial cells is attributed to a signaling pathway involving periostin/MMP-2, MMP-9, resulting in collagen Ⅰ production of airway fibroblasts. The process fuels the matrix stiffening [47]. The crosstalk of periostin and TSLP is an exquisitely driving factor for asthma [145]. In asthma patients, Kanemitsu et al. concluded that the accumulation of periostin in bronchial subepithelium was manifestly linked to the descent of FEV1 [146]. Anti-αMβ2 (specifically to periostin isoforms 1 and 8) and anti-ADAM8 blockers contained adhesion and migration of IL-5-stimulated eosinophils into periostin [113,147,148].

Serum periostin is linked to type 2 biomarkers, including eosinophilia, IgE concentration, and the fraction of NO (FeNO) inhalation, IL-4, and TSLP [149]. High serum periostin levels in patients receiving corticosteroids had prominent relation with the decline of pulmonary function tests and the increase of airflow limitation [46]. Both plasma periostin and saliva periostin levels had the advantage of early diagnosis of asthma [49]. Exhaled breath condensate (EBC) periostin levels seemed to reflect the emergence of CRS in asthma [54]. Sputum periostin levels offer an accurate diagnosis of serious asthma with continuous airflow limitation compared with mild-to-moderate asthma [55].

Lebrikizumab as an anti-IL-13 antibody was available to improve the function of the lung [150]. Periostin levels were strikingly correlated positively with the efficacy of these drugs, which included anti-IL-13 Ab-tralokinumab and dupilumab (common receptor of IL-4 and IL-13) [44,45]. The addition of dupilumab lowers serum periostin expression in AD, asthma, CRSwNP, and EE [26]. In addition to this, the effective therapeutic response of anti-IgE Ab omalizumab in asthma patients was dependent on high serum periostin [48]. Both hydroprednisone therapy and glucocorticoid-induced transcript 1 (GLCCI1) overexpression repressed the airway remodeling in asthma mice model via suppressing IL-13/periostin/TGF-β1 axis [50]. Clarithromycin can alleviate asthma by arresting periostin generation [53].

## 8. Lung Diseases

### 8.1. Pulmonary Fibrosis (PF)

Periostin is overexpressed in the lungs of patients with idiopathic pulmonary fibrosis (IPF). It was produced by fibroblasts and promoted their proliferation [151,152]. Nance et al. proposed that periostin mRNA was relatively lacking exon 21 in IPF samples compared to controls [115]. The absence of low-density lipoprotein receptor-related protein 1 (LRP1) prominently irritates the JNK/c-Jun/Fra-2 signaling pathway leading to the induction of α-SMA and periostin expression in human lung fibroblasts (hLF), tempting fibrosis of the lung [75]. Periostin furthered the recruitment of neutrophils and macrophages or myofibroblasts differentiation, accelerating pulmonary fibrosis [72,76]. The crosstalk of TGF-β and periostin also participated in the process of PF [73]. Serum monomeric periostin and EBC periostin both served as possible biomarkers to monitor IPF progression [74]. Moreover, serum periostin was also linked to fibrogenesis in COVID-19 [153]. Periostin of bronchoalveolar lavage fluid (BALF) might exaggerate the onset of eosinophilic pneumonia (EP), IPF, and COVID-19 [79,80]. The siRNA and antisense oligonucleotide targeting periostin, OC-20, and antibodies targeting αv integrin prevented lung fibrosis [151,154,155].

### 8.2. Pulmonary Hypertension (PH)

In ascending aortic constriction (AAC)-treated PH model, kcnk3-mutated rats presented greater expression of IL-6 and periostin in lung and heart as well as the lower extent of lung ctnnd1 mRNA levels, aggravating pulmonary and heart remodeling as well as lung vascular edema [77]. The feedback cycle between HIF-1α and periostin magnified PH by intensifying the proangiogenic role [78].

## 9. Atopic Dermatitis (AD)

Characteristics of AD include type 2 immune response, dermal fibrosis, barrier malfunction, and itch. Histamine and TNF superfamily member 14 (TNFSF14) upregulated periostin levels [156,157], which mediated the crosstalk of epithelial/mesenchymal. There exist two potential mechanisms to interpret it: first, IL-4/IL-13 tempts periostin secretion in fibroblasts. Periostin applies to keratinocytes via activating αv-mediated NF-κB signaling accompanied by the release of TSLP, which differentiates or stimulates DCs, developing a vicious cycle of type 2 inflammatory responses. Second, IL-1α and periostin are separately released by keratinocytes and fibroblasts, and their unity applies to fibroblasts by transducing the NF-κB pathway. Activated fibroblasts generate IL-6, contributing to the growth of keratinocytes. In addition, the cross-link of immune cells/non-immune cells with the help of periostin also accounted for the pathological mechanism of allergy. Periostin generated by fibroblasts amplifies adhesion, O2^−^ emergence, and TGF-β release in eosinophils. Activated eosinophils, in turn, lead to periostin generation in fibroblasts. Another paper uncovered the crosstalk of epithelial/sensory neurons. i.e., keratinocytes-derived TSLP directly targets TRPA1^+^ sensory neurons, irritating skin itch [36]. Signaling transmission of activating αvβ3/TRPV1/TRPA1/NPPB (natriuretic polypeptide B) in sensory neurons is involved in the periostin-mediated itch mechanism. The TSLP-periostin vicious loop also augmented inflammation and itch, creating ever-terrible circumstances: Keratinocytes secreted TSLP unlocking inflammatory response, and then TSLP back triggered the release of keratinocytes-derived periostin by means of TSLPR/JAK/STAT signaling propagation. In turn, periostin reciprocally stimulates the production of keratinocytes-derived TSLP. IL-13/STAT6/periostin/IL-24/STAT3 signaling transmission in keratinocytes sped up the inflammation process by incurring epidermal barrier malfunction [37].

The concentration of serum periostin rests on the grade of clinical severity of AD. It is related to other type 2 biomarkers―LDH and eosinophils, but not with IgE. Thus, monitoring it is of great help for the diagnoses and therapies of AD patients [158]. The blocking antibodies directed toward αv delayed AD progression [159]. By introducing dupilumab drugs, clinical outcomes were improved, and serum periostin evidently decreased [160]. Antioxidant cinnamaldehyde stimulated the NRF2/HMOX1 pathway and alleviated IL-13 and TGF-β1 mediated production of ROS, subsequently downregulating periostin in dermal fibroblasts. It may benefit in treating systemic fibrotic diseases [38].

## 10. Scleroderma

Periostin was upregulated in the skin of patients with scleroderma. The bleomycin-treated periostin^−/−^ mice showed reduced skin fibrosis followed by the descent of α-SMA^+^ myofibroblasts. However, recombinant mouse periostin resulted in the generation of collagen1α1 in myofibroblasts via the αv/PI3K/AKT signal axis [39]. Yamaguchi et al. discovered that periostin was colocalized with α-SMA^+^ myofibroblasts [161] and platelet endothelial cell adhesion molecule-1^+^ endothelial cells. Elevated serum periostin levels were associated with the severity of skin sclerosis. Crenolanib is an effective medication for diminishing skin and heart fibrosis by inhibiting periostin expression [40].

## 11. Eosinophilic Esophagitis (EE)

IL-13 and TGF-β stimulated periostin release in primary esophageal fibroblasts. Periostin was manifestly overproduced in the esophageal papillae and correlated positively with esophageal eosinophil amounts among patients with EE. The migration of eosinophils to the esophagus is due to the specific interaction of αM with periostin [41]. Elevated serum periostin levels were positively associated with IL-13 levels and may be used as a biomarker in EE in the presence of anti-IL-13 treatment [42].

## 12. Liver Diseases

In a mice model of dexamethasone (DEX)-treated fatty liver, DEX induced a higher degree of periostin expression in white adipose tissues, driving liver steatosis in a systemic organ-mediated fashion [81]. Periostin increased hepatic fibrosis and hepatic steatosis by inhibiting peroxisome proliferator-activated receptor-α(PPAR-α) expression [82]. Antisense oligonucleotides (ASOs) targeting periostin lowered hepatic steatosis in conjunction with reduced expression of α-SMA, collagen I, and other fibrotic markers and increased expression levels of PPAR-α. Another literature depicted that the periostin/α6β4/JNK/c-Jun prevented the binding of RORα to PPAR-α, suppressing PPAR-α expression and contributing to hepatosteatosis [83]. Periostin is mainly observed in activated hepatic stellate cells (HSCs). Periostin tempted liver fibrosis by activating LOX and lysyl oxidase-like (LOXL) in chronic liver disease via the αvβ3/PI3K/Smad2/3 signaling pathway [85]. Periostin deletion devastated angiogenesis in the process of liver regeneration [86]. Serum periostin is forcefully correlated with higher nonalcoholic fatty liver disease (NAFLD) [84].

## 13. Chronic Kidney Disease (CKD)

Periostin is overexpressed in a variety of kidney diseases. It is mainly presented in the glomerulus, renal arteries, tubular cells, and interstitial area. For healthy donors, periostin is found in the vascular pole of the glomerulus and around Bowman’s capsule. Some opposite evidence confirmed that periostin has no expression in healthy kidney specimens. Periostin upregulation contributed to the fibrosis of CKD disease by inducing the FAK/p38/ERK pathway and expression of collagen I [92]. Periostin strengthened fibrosis and apoptosis in tubular epithelial cells by activating the phosphorylated-p38 MAPK pathway, facilitated vascular calcification through αvβ3/Wnt/β-catenin signaling, and accelerated inflammatory reaction by activating the β3/FAK/AKT pathway under NF-κB medication or mTOR complex 1 (mTORC1)-mediated inhibition of autophagy in CKD [87,88,93,94]. Additionally, periostin/αv/ILK (integrin-linked kinase) and periostin/αvβ3/AKT/mTOR signaling pathways both aggravated the growth of cyst epithelial cells in autosomal dominant polycystic kidney disease (ADPKD) [5,89]. In contrast to the above reporter, periostin served beneficial roles in renal repair, such as driving the proliferation of tubular cells via binding to integrin-β1 as well as the polarization of macrophage embodying pro-reparative characteristic following acute kidney injury (AKI) [95].

In hypertensive nephropathy, periostin correlated positively with creatinine and proteinuria. Losartan deterred periostin synthesis leading to lower renal fibrosis [90]. In diabetic renal disease, elevated urine periostin content was accompanied by the emergence of albuminuria [162]. Moreover, serum periostin could estimate diabetic disease stages [97].

Periostin advanced the proliferation of mouse mesangial cells (MMCs) to augment renal malfunction in Immunoglobulin A nephropathy (IgAN) [96]. Urine periostin concentration correlated with tissue fibrosis in biopsy-proven IgA nephropathy subjects [163]. During the progression of UUO, mechanical stress as an initiating signal increased periostin accumulation in collecting duct cells. Subsequently, periostin advanced the production of proinflammatory factor MCP-1 that mediated macrophage infiltration, and then TGF-β secreted by infiltrating cells induced periostin production and strengthened the phenotype change of tubular epithelial cells [92]. After 5/6 nephrectomy, periostin which was detected in the distal tubule (DT) epithelial cell, drove the expression of fibroblast-specific protein-1 (FSP-1) and MMP-9 in distal collecting tubular cells [91]. Platelet-derived growth factor-BB (PDGF-BB) stimulated the PI3K/AKT/periostin signaling cascade, driving the expression of fibronectin and proliferation in MMCs in lupus nephritis [164].

## 14. Inflammatory Bowel Disease (IBD)

Periostin and αv integrin are more strongly presented in the colon tissues of UC (Ulcerative colitis) patients than in healthy colonic mucosa. Periostin accumulation occurred in pericryptal fibroblasts [165]. Introducing recombinant periostin elicits colitis in periostin-absence mice, and the blocking antibody specific to periostin obviously mitigates intestinal inflammatory disease. TNF-α stimulates the expression of periostin mRNA in intestinal epithelial cells (IECs). Periostin induced IL-8 expression and magnified NF-κB activity in IECs. Meanwhile, the combination of periostin with TNF-α synergistically reinforced IL-8 levels via interaction with integrin αv [98]. The pIBD patients presented elevated peri-cryptal staining compared to controls, but the expression pattern of periostin isoforms showed no significance. Thus, certain specific periostin isoforms and changes in periostin-binding molecule expression levels in the peri-cryptal ring might account for enhanced pericryptal periostin rings in pediatric IBD (pIBD) patients. Great plasma levels of the periostin during the period of pIBD remission may participate in mucosal healing and tissue repair [99]. Another literature on Crohn’s disease (CD) ascertained the cut-off levels of serum periostin in adult patients to serve to diagnose CD and forecast the activity status of CD [100].

## 15. Osteoarthrosis

### 15.1. Rheumatoid Arthritis (RA)

In a mouse model of mocking arthritis, periostin loss mice appeared to have a higher degree of inflammation. In RA remission, serum periostin embodied high extent of levels [104], increasing the risk of fragility fractures.

### 15.2. Osteoarthritis (OA)

Normal articular chondrocytes highly expressed isoforms 1 and 5, and anterior cruciate ligament (ACL) progenitor cells overexpressed isoforms 3, 4, 6, 7, and 8. ACL progenitor cells that highly expressed total periostin, not isoform 1, showed higher cell adhesion than articular chondrocytes that expressed lower total periostin [117]. Mechanical pressure, as the primary reason, initiates and fuels inflammatory responses of OA. The cDNA array analysis revealed that periostin is at maximal levels in the cartilage of OA than controls. The periostin-positive signal was detected in chondrocytes, periphery matrices close to the degraded region, fibrotic cartilage, and tissue of subchondral bone. The application of periostin into isolated human chondrocytes might provoke a high expression of IL-6 and IL-8 accompanied by the sufficient expression of MMP-1, MMP-3, MMP-13, and nitric oxide synthase-2(NOS2) in an NF-κB-activated mechanism [105]. Periostin accelerated cartilage denaturation in Wnt/β-catenin/MMP-13/ADAMTS4- or discoidin domain receptor-1(DDR1)/Akt/Wnt/β-catenin/MMP-13-dependent mechanism [106]. It also had a contributory effect on MMP-2 and MMP-3 expression in OA synoviocytes. Synovial fluid (SF) periostin was positively associated with the progression of OA [109].

### 15.3. Ankylosing Spondylitis (AS)

Periostin was secreted by osteoblasts in AS. Serum periostin was higher under high inflammatory factors, disease severity, and low radiographic injury conditions [166].

### 15.4. Osteoporosis

Periostin lowers sclerostin levels, followed by the activation of the LRP5/Wnt/β-catenin cascade, boosting gene transcription within osteoblasts to induce bone formation [101]. In huRANKL-overexpressed mice, cathepsin K (Ctsk) limited bone formation and increased bone fragility by preventing periostin generation, which offers an underlying mechanism for osteoporosis in PMW [167]. The 17β-E2/periostin/Wnt/β-catenin pathway can enhance the osteogenesis of bone marrow stromal cells (BMSCs) in ovariectomized (OVX) rats, thereby decreasing osteoporosis [102]. Periostin also reinforced the osteogenic competence of bone marrow skeletal stem cells in an ILK/Akt/GSK-3β-activated manner [103]. Serum periostin is related inversely to bone mineral density (BMD) in Chinese postmenopausal women (PMW) [168]. Cathepsin K-generated periostin (K-Postn) predominantly reflected fracture of Caucasian PMW with primary hyperparathyroidism (PHPT) [110].

### 15.5. Developmental Dysplasia of the Hip (DDH)

In chondrocytes, periostin upregulated IL-6 and MMP-3 levels based on the integrin-FAK-Src-NF-κB pathway. Meanwhile, it limited the production of Col2a1 and Acan. Then, IL-6/STAT3/periostin and MMP-3, as a vicious feedback loop, augmented hip dislocation-induced acetabular cartilage denaturation [107].

### 15.6. Intervertebral Disc Degeneration (IVDD)

Periostin accelerated nucleus pulposus cells (NPCs) apoptosis and intervertebral disc denaturation via the Wnt/β-catenin pathway [108].

## 16. Conclusions

Periostin exerts an integral role in the crosstalk between tumor cells and tumor microenvironments, cell and matrix, physiological function, and pathological function. Although the substantial data proved its significance in tissue remodeling, fibrosis, inflammation, wound healing, repair, and vascular calcification mediated by diverse signaling pathways, there were still a few works that determined its protective roles in ameliorating CRS and asthma, promoting the regeneration of myocardium and liver as well as renal repair, and maintaining periodontal stabilization, these discrepancies are most probably due to differences in animal models. In addition, different roles caused by disparate diseases are likely attributed to different locations, cell types that respond, and pathologic processes of these diseases. Periostin serves functions by diverse signaling pathways such as FAK, Src, NF-κB, p38, ERK, mTOR, JNK, PI3K, Akt, Smad2/3, MAPK, Wnt/β-catenin, to name just a few. Thus, the application of therapies based on periostin function is of great account and creates a favorable outlook for subsequent clinical studies.

It should be noted that loss of the αv integrin as a way of blocking periostin gives play to the majority of undesirable accidents such as prenatal death, colitis, wasting, and autoimmunity [169,170]. We still need to make significant efforts to boost the development of precision medicine through current knowledge and continuous explorations on exact and detailed mechanisms of periostin-involved diseases, despite the journey being full of challenges.

Furthermore, periostin exhibited the potential of acting as a clinically relevant and serviceable biomarker to aid in the diagnosis, speculate on the progression and activity of the disease, inform on prognosis, and direct choice for therapeutic approaches of disease. Periostin, as an attractive and available biomarker for inflammatory diseases, is presently garnering extensive attention. Nevertheless, it must be noticed that the flaw of periostin as a biomarker is that basal expression levels of serum periostin are held high in childhood until bone development halts [171]. Another issue is that periostin isoforms (1 or 2, 3, 4, 5, and 6) and IgA form a complex in serum, which possibly influences the measuring of serum periostin [118].

Different tissues are characterized by heterogeneous expression profiles of periostin isoforms. Currently, the pathological roles of only several periostin isoforms have been displayed, and the functions of each encoded isoform have not been entirely exposed. Further exploration is needed to analyze the functional property of each coding isoform. Moreover, it is of great urgency to develop emerging drugs on the basis of the stimulators or inhibitors affecting periostin expression, periostin itself, the periostin-involved receptors and signaling pathways, or certain periostin isoforms-mediated channels.

## Figures and Tables

**Table 1:** Expression (upregulation **↑** or downregulation ↓) and roles of periostin, the periostin-involved signaling pathways, therapies based on periostin, and potential disease biomarkers in disease progression.

Tissues/ Diseases	Expression of Periostin	Roles of Periostin	Reference	Periostin- Involved Downstream Signaling Pathways	Reference	Therapies Based on Periostin	Reference	Potential Disease Biomarkers	Reference
EBI and cerebral ischemia	↑	BBB disruption	[11]	p38/ERK/MMP-9	[11]	IAXO-102 Clarithromycin	[12] [13]	Serum periostin	[14]
Ocular diseases	↑	Inflammation	[15]			Betamethasone; tacrolimus	[16]	Tear periostin	[17]
		Tissue remodeling	[18]					Serum periostin	[19]
CRS and AR	↑	Inflammation	[20,21]			Omalizumab; mepolizumab; methylprednisolone; doxycycline	[22]	Serum periostin	[23]
		Tissue remodeling	[21,24]	Src/AKT/mTOR	[24]				
		Protective role	[25]			Dupilumab	[26]	NLF periostin	[27]
						GCs	[28]		
						AH	[29]		
						AG-Ex	[30]		
Periodontitis	↑	Tissue remodeling	[31]					GCF periostin	[32,33]
		Wound healing	[31]	β1/FAK/JNK; β1/RhoA	[31]				
		Bone formation	[34,35]					Saliva periostin	[33]
AD	↑	Inflammation	[36]	αv/NF-κB/TSLP; NF-κB/IL-6	[36]	Dupilumab	[26]		
		Itch	[36]	αvβ3/TRPV1/TRPA1/NPPB; TSLP/TSLPR/JAK/STAT	[36]				
		Epidermal barrier malfunction	[37]	IL-24/STAT3	[37]	Cinnamaldehyde	[38]		
Scleroderma	↑	Skin fibrosis	[39]	αv/PI3K/AKT/collagen1α1	[39]	Crenolanib	[40]	Serum periostin	[40]
EE	↑	Inflammation	[41]	αM	[41]	Dupilumab	[26]	Serum periostin	[42]
Asthma	↑	Inflammation	[43]			Tralokinumab; dupilumab	[44,45]	Serum periostin	[46]
		Tissue remodeling	[47]			Omalizumab	[48]	Plasma periostin	[49]
						Hydroprednisone	[50]	Saliva periostin	[49]
		Protective roles	[51,52]			Clarithromycin	[53]	EBC periostin	[54]
								Sputum periostin	[55]
Cardiovascular diseases (MI, heart failure, VHD, hypertension, and vascular calcification)	↑	Inflammation	[56]	Periostin/NLRP3/caspase-1	[56]	Valsartan	[57]	Plasma periostin	[58]
		Tissue remodeling	[59,60,61,62,63,64]						
		Wound healing	[59,65]			Simvastatin	[63]		
		Cardiomyocytes apoptosis	[66]						
		Myocardial regeneration	[5,67,68]	αvβ1/αvβ3/αvβ5/PI3K/Akt; TNF-α/NF-κB; PI3K/AKT/cyclin D1	[5,67,68]	Resveratrol	[69]		
		Angiogenesis	[5]						
		Vascular calcification	[70,71]			Crenolanib	[40]		
Lung diseases (PF, EP, COVID-19, and PH)	↑	Inflammation	[72,73]					Serum monomeric periostin	[74]
		Tissue remodeling	[72,75,76,77]					EBC periostin	[74]
		Angiogenesis	[78]					BALF periostin	[79,80]
Liver disease	↑	Liver steatosis	[81,82,83]	α6β4/JNK/c-Jun	[83]			Serum periostin	[84]
		Hepatic fibrosis	[82,85]	αvβ3/PI3K/Smad2/3/LOX/LOXL	[85]				
		Liver regeneration and angiogenesis	[86]						
CKD	↑	Inflammation	[87,88]	αv/ILK; β3/FAK/AKT; αVβ3/AKT/mTOR	[5,88,89]	Losartan	[90]	Urine periostin	[91]
		Renal fibrosis	[87,92,93]	FAK/p38/ERK; p38 MAPK	[92,93]				
		Vascular calcification	[94]	αvβ3/Wnt/β-catenin	[94]				
		Renal repair	[95]						
		Renal malfunction	[96]					Serum periostin	[97]
IBD	↑	Inflammation	[98]	NF-κB; αv/IL-8	[98]			Plasma periostin	[99]
								Serum periostin	[100]
Osteoarthrosis (RA, OA, AS, osteoporosis, DDH, and IVD D)	↓ in RA and osteoporosis	Bone formation	[101,102,103]	Inhibition of sclerostin/LRP5/Wnt, β-catenin; Wnt/β-catenin; ILK/Akt/GSK-3β;	[101,102,103]			Serum periostin	[104]
	↑ in OA, AS, DDH, and IVDD	Inflammation	[105,106,107,108]	NF-κB/IL-6/8; Wnt/β-catenin/MMP-13/ADAMTS4; DDR1/Akt/Wnt/β-catenin/MMP-13; αvβ3/FAK/Src/NF-κB/IL-6/STAT3	[105,106,107,108]			SF periostin	[109]
								K-Postn	[110]

**Table 2:** The expression and roles of periostin isoforms in tissues/diseases.

Tissues/Diseases	Certain Periostin Variants Expressed in Tissues/Diseases	Roles of the Periostin Variants	Reference
Cerebral ischemia	Isoform 2	Isoform 2 Minimizing the area of cerebral infarction via phosphorylation of Akt	[111]
Asthma	Isoforms 6, 7, and 8	Isoform 8 Promoting the eosinophil adhesion under IL-5 stimulation αMβ2]	[112,113]
MI	Isoforms 1, 2, 5, and 6	Isoform 1 Decreasing the attachment of fibroblasts and myocytes as well as facilitating myocytes death leading to ventricular dilation and tissue remodeling Isoform 6 Contributing to the migration of activated fibroblasts and healing of impaired tissue via the αv/FAK/AKT signaling pathway	[114]
IPF	All periostin variants lacking exon 21	-	[115]
Retinal ischemia	Isoforms 1, 2, and 5	Isoforms 1, 2, and 5 Promoting preretinal pathological NV	[116]
pIBD	Isoforms 2, 6, 7, and 8	-	[99]
Joint	Articular chondrocytes highly expressed isoforms1 and 5, and anterior cruciate ligament(ACL) progenitor cells overexpressed isoforms 3, 4, 6, 7, and 8	-	[117]
Serum	At least five isoforms, including 1 or 2, 3, 4, 5, 6	At least five isoforms, including 1 or 2, 3, 4, 5, 6 Forming complex with IgA	[118]

## Data Availability

All cited articles in the current study are available in the public database.

## Abbreviations

OSF-2	Osteoblast-specific factor-2
FAS1	Fasciclin I
HDFs	Human dermal fibroblasts
PDL	Periodontal ligament
FAK	Focal adhesion kinase
GCF	Gingival crevicular fluid
AgP	Aggressive periodontitis
CRS	Chronic rhinosinusitis
AR	Allergic rhinitis
CRSwNP	CRS with nasal polyposis
GCs	Glucocorticoids
CT	Computed tomography
TSTP	Thymic stromal lymphopoietin
NLF	Nasal lavage fluids
SLIT	Sublingual immunotherapy
AH	Asarum heterotropoides
HNEpCs	Human nasal epithelial cells
AG-Ex	Angelica gigas extract
CFs	Cardiac fibroblasts
MI	Myocardial infarction
MAPK	Mitogen-activated protein kinase
CREB	Cyclic AMP response element-binding protein 1
VSMCs	Vascular smooth muscle cells
LOX	Lysyl oxidase
BMP-1	Bone morphogenic protein-1
Snhg8	Small nucleolar RNA host gene 8
NMCMs	Neonatal mouse cardiomyocytes
MSCs	Mesenchymal stem cells
EAM	Experimental autoimmune myocarditis
AF	Atrial fibrillation
GSN	Gelsolin
FMOD	Fibromodulin
MIRI	Myocardial ischemia-reperfusion injury
SIM	Simvastatin
VHD	Valvular heart disease
VICs	Valvular interstitial cells
MMP-2	Matrix metalloproteinase-2
ANP	Atrial natriuretic peptide
AHR	Airway hyperresponsiveness
Th2	T-helper type 2
GCM	Goblet cell metaplasia
HDM	House dust mite
DCs	Dendritic cells
EBC	Exhaled breath condensate
GLCCI1	Glucocorticoid-induced transcript 1
EE	Eosinophilic esophagitis
AD	Atopic dermatitis
TNFSF14	TNF superfamily member 14
NPPB	Natriuretic polypeptide B
BP	Bullous pemphigoid
SD	Stasis dermatitis
IBD	Inflammatory bowel disease
UC	Ulcerative colitis
CD	Crohn’s disease
pIBD	Pediatric IBD
IECs	Intestinal epithelial cells
PH	Pulmonary hypertension
AAC	Ascending aortic constriction
RA	Rheumatoid Arthritis
OA	Osteoarthritis
ACL	Anterior cruciate ligament
NOS2	Nitric oxide synthase-2
DDR1	Discoidin domain receptor-1
SF	Synovial fluid
AS	Ankylosing Spondylitis
Ctsk	Cathepsin K
BMSCs	Bone marrow stromal cells
OVX	Ovariectomized
BMD	Bone mineral density
PMW	Postmenopausal women
K-Postn	Cathepsin K-generated periostin
PHPT	Primary hyperparathyroidism
DDH	Developmental dysplasia of the hip
IVDD	Intervertebral disc degeneration
NPCs	Nucleus pulposus cells
AC	Atopic conjunctivitis
AKC	Atopic keratoconjunctivitis
FADS	Facial atopic dermatitis with scratching
PVR	Proliferative vitreoretinopathy
FVM	Fibrovascular membrane
NV	Neovascularization
IPF	Idiopathic pulmonary fibrosis
LRP1	Low-density lipoprotein receptor-related protein 1
hLF	Human lung fibroblasts
BALF	Bronchoalveolar lavage fluid
EP	Eosinophilic pneumonia
CAC	Coronary artery calcification
PPAR	Peroxisome proliferation-activated receptor
EBI	Early Brain Injury
BBB	Blood-brain barrier
SAH	Subarachnoid hemorrhage
TLR4	Toll-like receptor 4
DEX	Dexamethasone
ASOs	Antisense oligonucleotides
HSCs	Hepatic stellate cells
LOXL	Lysyl oxidase-like
NAFLD	Nonalcoholic fatty liver disease
CKD	Chronic kidney disease
UIRI	Unilateral ischemia-reperfusion injury
AKI	Acute kidney injury
mTORC1	mTOR complex-1
ILK	Integrin-linked kinase
ADPKD	Autosomal dominant polycystic kidney disease
PA	Periostin-binding DNA aptamer
IFTA	Interstitial fibrosis/tubular atrophy
MCs	Mesangial cells
IgAN	Immunoglobulin A nephropathy
DT	Distal tubule
FSP-1	Fibroblast specific protein-1
RAAS	Renin-angiotensin-aldosterone system
PDGF-BB	Platelet-derived growth factor-BB
PCNA	Proliferating cell nuclear antigen
MMCs	Mouse mesangial cells
